# Feasibility of complex exercise therapy with Standing Ovation and peripheral neuromodulation for gait rehabilitation after incomplete spinal cord injury—a case report

**DOI:** 10.1007/s10354-024-01046-8

**Published:** 2024-06-05

**Authors:** Anna Pataraia, Winfried Mayr, Jose Luis Vargas Luna, Julia Sternik, Richard Crevenna

**Affiliations:** 1 Department of Physical Medicine, Rehabilitation and Occupational Medicine, Medical University of Vienna, Vienna, Austria; 2 Center for Medical Physics and Biomedical Engineering, Medical University of Vienna, Vienna, Austria; 3 Department of Physical Medicine, Rehabilitation and Occupational Medicine, Medical University of Vienna, Währinger Gürtel 18–20, 1090 Vienna, Austria

**Keywords:** Spinal cord injury, Exercise therapy, Standing Ovation, Neuromodulation

## Abstract

**Background:**

Spinal cord injuries (SCIs) are a global concern, annually affecting hundreds of thousands of individuals. Among these cases, incomplete SCIs, allowing some muscle activity below the injury, pose unique challenges. This case study focuses on a 55-year-old male with a moderate incomplete SCI (AIS-D).

**Case presentation:**

After initial treatments and pharmaceutical antispastic therapy, a novel intervention was introduced featuring the Standing Ovation gait exercise system (Standing Ovation GmbH, Hallwang, Austria). This individually tailored system, equipped with a rail system and seat-lifting unit, provided a secure environment for balance training. Over 14 training sessions spanning from October 13, 2021, to March 23, 2022, improvements in functional walking were observed.

**Discussion and conclusion:**

Locomotor improvement in SCI rehabilitation is important; the potential of task-specific gait exercises with the Standing Ovation system in incomplete spinal cord injury seems to be a promising approach. Although promising, these findings call for further systematic studies with larger patient cohorts to strengthen their reliability. Ongoing research endeavors are essential to fully understand the benefits and limitations of this intervention in spinal cord injury rehabilitation.

## Background

Spinal cord injuries (SCIs) frequently lead to mobility constraints, reduced physical activity levels, and, consequently, a diminished quality of life [1, 2]. Every year, an estimated 250,000 to 500,000 new SCIs occur globally [1]. Approximately one third of these cases involve incomplete lesions, allowing for some muscle activity below the affected area [3]. In comparison to complete injuries, incomplete SCIs tend to be less severe, although patients still encounter limitations in various aspects of their lives, with an increased vulnerability to falls [4–7].

The most conspicuous sign of SCI is impaired locomotion, often accompanied by additional medical complications such as an elevated risk of fractures. Recognizing the complex nature of these challenges, efforts to enhance quality of life for individuals with incomplete or complete SCIs have evolved over time. The focus has shifted from merely extending life expectancy to facilitating independent living and an optimal quality of life.

**Fig. 1:**
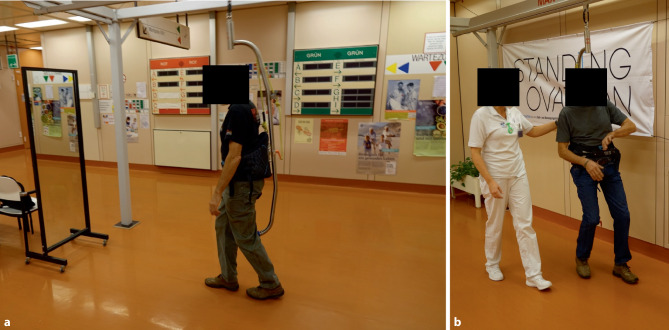
**a**, **b** Exercise therapy in Standing Ovation (Standing Ovation GmbH, Hallwang, Austria). (Patient receives tailored physical therapy with simultaneous electrical stimulation to improve gait and reduce spasticity)

In this context, we present a case study of a patient with a moderate incomplete SCI (AIS-D). This individual engaged in task-specific overground gait exercises aimed at promoting locomotor improvement (Fig. 1). These exercises hold promise as a therapeutic approach to address the challenges posed by SCI and enhance walking ability in individuals with incomplete injuries.

## Case presentation

A 55-year-old male with an incomplete SCI below C6 after a diving injury (acute decompression syndrome) underwent initial treatment with decompression therapy and inpatient spinal cord rehabilitation. Two years later he was referred to the Department of Physical Medicine, Rehabilitation and Occupational Medicine, Medical University of Vienna, Austria, for spinal cord stimulation for spasticity reduction. At the time of first presentation, the patient had reduced ambulatory performance and endurance, muscle rigidity, reflex hypertonia, peroneal paresis on both sides, mild spasticity of the legs, and bladder dysfunction. His gait was impaired due to mild spasticity while walking as well as peroneal paresis. Since the very beginning of the injury, the patient had had antispastic treatment with baclofen 75 mg/d. Brain motor control assessment (BMCA) was performed, and the patient was instructed for afferent stimulation, but the home program was not regularly carried out. BMCA is a method to record electrical activity from selected muscles through surface electromyography (EMG) during the performance or attempted performance of volitional and reflex motor tasks [8]. It is used to characterize impaired motor control below the lesion and also to quantify changes induced by clinical interventions. As spasticity was not severe, the patient was asked to slowly taper and stop baclofen, and the intervention with the Standing Ovation stance and movement support system (Standing Ovation GmbH, Hallwang, Austria) was started. Standing Ovation is an individually adapted rail system with a seat-lifting unit, which allows a three-dimensional movement pattern, reduces the additional strain on the legs, and prevents fall risks during exercising. Fourteen training units were performed with interruptions (due to the pandemic lockdowns and strict testing regulations) between October 13, 2021, and March 23, 2022. In addition, a subthreshold afferent stimulation of the peroneal nerve was applied using a Cefar Rehab X2 stimulator (DJÖ FRANCE, Mouguerre, France) and bilateral hydrogel stimulation electrodes (Axion GmbH, Leonberg, Germany), one with a 3.2 cm round electrode (anode first phase) placed over the peroneal nerve proximally to the lateral fibular head, and a counter electrode 50 × 50 mm positioned at the proximal third of the tibialis anterior muscle. The stimulator delivered continuous trains of biphasic rectangular charge-balanced pulses with a phase duration of 400 µs and a frequency of 30 Hz. Intensity was adjusted to a slightly subsensory threshold. The patient applied stimulation during overground gait episodes in both in-home and wildlife environments for up to 2 h, but not on a consequently documented regular basis. Exercising secured by the Standing Ovation device provided secure conditions for complex balance skill exercising and showed promising developments in terms of restoring functional walking, which enabled him to maintain a healthy lifestyle, reduce the fall risk, and increase his level of physical activity. The patient reported improvements in activities of daily living, in particular regarding improvement of walking distance on various surfaces, and increased endurance during hiking, which was limited previously.

## Discussion and conclusion

The intervention approach described herein presents a versatile array of delivery methods encompassing options such as robotic-assisted training overground or treadmill training—either with or without body support and accompanied by manual step or electrical stimulation assistance—alongside the more traditional overground gait training [9–14]. Extensive research has already emphasized the advantageous impact of high-frequency repetitive locomotor training in fostering the resurgence of typical gait patterns post-SCI [10, 12]. Given the paramount significance of regaining walking proficiency in the rehabilitation journey of SCI patients, the exploration of locomotor training as an intervention strategy for tackling gait limitations within this population has been met with thorough investigation and keen interest [9, 13–16].

Against this backdrop, the application of task-specific gait exercises, facilitated by the innovative Standing Ovation system, has exhibited potential to significantly contribute to locomotor recovery, particularly in cases of moderate incomplete SCI. By offering a secure platform for gait enhancement, the Standing Ovation system creates an environment conducive to fostering substantial strides in locomotion recuperation.

However, while the initial results are promising, a comprehensive understanding necessitates the undertaking of systematic studies encompassing a broader patient cohort. By delving into a more extensive sample size, the reliability and generalizability of the observed outcomes can be fortified, reinforcing the efficacy of the task-specific gait exercises in tandem with the Standing Ovation system. These future investigations stand to provide a more comprehensive perspective on the therapeutic benefits and potential limitations of this intervention strategy.

In conclusion, the fusion of task-specific gait exercises with the adaptable Standing Ovation system introduces an encouraging avenue for augmenting locomotor recovery in the context of moderate incomplete SCI.
